# Common Acquisition of Broadly Neutralizing Antibodies in an HTLV-1c+ First Nations Cohort from Central Australia

**DOI:** 10.3390/v18040402

**Published:** 2026-03-24

**Authors:** Samantha L. Grimley, Sarah C. Monard, Ashley Hirons, Ashley H. Y. Yap, Sarah Collins, David Yurick, Georges Khoury, Paula C. Ellenberg, Marc Pellegrini, Lloyd J. Einsiedel, Damian F. J. Purcell

**Affiliations:** 1 Department of Microbiology and Immunology, The Peter Doherty Institute for Infection and Immunity, The University of Melbourne, Melbourne, VIC 3000, Australia; 2 Département de Biologie, École Normale Supérieure de Lyon, Université Claude Bernard Lyon I, Université de Lyon, 69342 Lyon, France; 3 UCB Pharma, Smyrna, GA 30080, USA; 4 Centre for Virology, Burnet Institute, Melbourne, VIC 3004, Australia; 5 Division of Infectious Diseases and Immune Defence, The Walter and Eliza Hall Institute of Medical Research, Parkville, VIC 3050, Australia; 6 Department of Medical Biology, The University of Melbourne, Parkville, VIC 3010, Australia; 7 Centenary Institute of Cancer Medicine and Cell Biology, Camperdown, Sydney, NSW 2050, Australia; 8 Department of Medicine, Alice Springs Hospital, Alice Springs, NT 0870, Australia

**Keywords:** HTLV-1, proviral load, Envelope, seroprevalence, neutralizing antibodies

## Abstract

Human T-cell leukemia virus type-1 (HTLV-1) is endemic to numerous regions worldwide, including Central Australia. The Australo-Melanesian subtype-C is endemic within Australia and Oceania, whereas subtype-A is the most widely distributed subtype globally. The lack of an approved vaccine highlights HTLV-1 as a neglected public health issue. To inform the development of HTLV-1 Envelope (Env)-based vaccines, we assessed anti-Env antibodies in an HTLV-1c+ cohort of First Nations individuals in Central Australia. Of the 62 plasma samples from patients with confirmed HTLV-1 serological diagnosis, 76% were positive for Env binding in ELISA, but 90% neutralized HTLV-1c pseudovirus (PSV) infection. Neutralization breadth with the capability of blocking both subtype-A and subtype-C PSV infection was identified in 100% of samples tested. Proviral load was positively associated with anti-Env response, with binding epitopes mapping to the proline-rich region of gp46-SU. Env-directed IgG showed the capacity to engage Fcγ receptors key to inducing antibody-dependent cellular cytotoxicity/phagocytosis responses. Serological response was not associated with comorbidities linked to HTLV-1c in this population (bronchiectasis, chronic kidney disease, diabetes). These findings demonstrate that potent humoral immunity arises and is sustained during HTLV-1 infection, suggesting that an Env-based vaccine displaying authentically native epitopes will be capable of recapitulating these neutralizing responses.

## 1. Introduction

Human T-cell leukemia virus type 1 (HTLV-1) is an oncogenic human deltaretrovirus endemic to numerous regions worldwide, including Japan, the Caribbean islands, South America, Central and West Africa, Romania, the Middle East and Central Australia [1]. An estimated 10–20 million individuals are currently infected worldwide, with Australia harboring some of the highest recorded infection rates in remote First Nations communities, where up to 40% of adults have tested positive for HTLV-1 [2].

HTLV-1 is the etiological agent for adult T-cell leukemia/lymphoma (ATL) and HTLV-1-associated myelopathy (HAM), which arise in 5% and 3% of cases, respectively [3,4]. In Australia, infection is also associated with inflammatory diseases such as bronchiectasis, infective dermatitis, uveitis, chronic kidney disease (CKD) and diabetes [5,6]. HTLV-1 is predominantly transmitted by sexual intercourse, blood–blood transfer or mother-to-child transmission (MTCT), primarily via breastfeeding [7,8]. Virus budding at the infected host cell surface is mostly trapped in a biofilm, so transmission occurs almost exclusively by infected cell-to-cell contact, although a low level of transmission can occur by infrequent cell-free viral particles [9,10,11,12]. HTLV-1 causes lifelong infection, and there are currently no approved therapeutic treatments or vaccines for the treatment or prevention of HTLV-1 infection [13].

There are seven subtypes of HTLV-1 circulating worldwide (A–G), of which the Australo-Melanesian HTLV-1c subtype is endemic within Australia, Papua New Guinea, the Solomon Islands and Vanuatu [14]. HTLV-1a is the most widely distributed subtype globally and hence, the most studied [1]. The early evolutionary divergence of HTLV-1c has led to high genetic stability and low sequence variation within the subtype, and comparison of structural proteins such as the Envelope (Env) glycoprotein between subtypes A and C shows sequence similarity at the amino acid (aa) level of between 94 and 97% [15,16,17].

The HTLV-1 Env glycoprotein is the sole viral structural protein expressed and displayed on the external surface of the virion/infected cell and, as such, has been the primary focus of prophylactic vaccine design strategies [18]. Env-targeting antibodies arising during HTLV-1a infection have demonstrated neutralizing activity in cases of ATL and HAM. However, antibody (Ab) levels did not correlate with disease, so they are unlikely to serve as a predictive biomarker of disease severity or be useful as a therapeutic in infected individuals to prevent disease [19,20].

Env is synthesized as a 62 kDa precursor that is cleaved by cellular furin-like proteases in the Golgi apparatus to produce the surface (SU) and transmembrane (TM) subunits gp46 and gp21, respectively. The gp46 and gp21 subunits assemble as a trimer of heterodimers on the viral or infected cell membrane. HTLV-1 infection occurs by interaction of the Env trimer with the reported host cell receptors glucose transporter type 1 (GLUT1), heparin sulphate proteoglycans (HSPGs) and neuropilin 1 (NRP1) [21,22,23,24]. Host cell entry can be blocked by Env-directed neutralizing antibodies (nAbs), with multiple epitopes being identified as common sites of vulnerability during HTLV-1a infection. These include two N-terminal regions located between residues 53–75 and 86–107 [25], residues 175–199 located within the proline-rich region (PRR) [26,27] and residues 287–311 located within the C-terminal regions [28]. Numerous monoclonal antibodies (mAbs) have been reported that target the PRR, including LAT-27, a nAb of rat origin with a well-conserved binding epitope between residues 191 and 196 that has demonstrated protective efficacy in a humanized mouse model [29,30,31]. Many of these neutralizing epitopes are conserved between subtypes A and C, suggesting that the development of biotherapeutics incorporating these epitopes, such as native-like Env-based vaccines and Env-directed mAb treatments, could universally prevent against infection.

Whilst the neutralizing activity of a vaccine-induced humoral response is important in the success of an antiviral response in vivo, Fc-mediated effector functions, such as antibody-dependent cellular cytotoxicity (ADCC) and phagocytosis (ADCP), can also be vital in killing and clearing virus-infected cells [32,33]. Antiviral activity is mediated by the engagement of the Fc segment of an immunoglobulin with Fc receptors present on different immune cells, therefore independent of neutralizing ability [34]. Monoclonal antibodies isolated from HTLV-1-infected and immunized animals, as well as antiserum from HTLV-1+ individuals, have been reported to exhibit ADCC [26,35], yet the role of Fc-mediated effector functions during natural infection in vivo remains unclear.

Although GLUT1, HSPG and NRP1 are constitutively expressed, HTLV-1 preferentially infects CD4+ T cells, whereby the (+) ssRNA genome is converted into dsDNA and integrates randomly into the host genome as a provirus, establishing life-long infection. The measurement of integrated proviral DNA, or proviral load (PVL), is a demonstrated correlate of disease severity in HAM [36,37] and bronchiectasis [5] and a strong correlate of Ab titer in HTLV-1a+ individuals [38].

In this study, we examined the antiviral activity of the serological response to Env during natural infection in an HTLV-1c+ First Nations cohort.

## 2. Materials and Methods

### 2.1. Ethics Statement

Human whole blood was donated by serologically diagnosed persons with HTLV-1c infection and uninfected individuals at Alice Springs Hospital, Northern Territory, after written consent in their primary languages. This study was conducted in accordance with the National Health and Medical Research Council (NHMRC) of Australia guidelines and approved by the Central Australian Human Research Ethics Committee (CAHREC) (HREC 14249). Clinical information for this hospital-based cohort is summarized in Table 1.

### 2.2. Sample Preparation

Donor peripheral blood mononuclear cells (PBMCs) and plasma were isolated from whole blood using Ficoll density gradient centrifugation (Ficoll 400, F4375, Merck, Bayswater, Australia) at the Baker Institute, Alice Springs, and stored in liquid nitrogen. Samples were sent to the Doherty Institute at the University of Melbourne for processing and analysis.

### 2.3. HTLV-1 Serologic and Molecular Studies

Diagnosis of HTLV-1 serostatus was based on the detection of specific anti-HTLV-1 antibodies in serum by enzyme immunoassay (EIA) (Murex HTLVI+II; DiaSorin, Saluggia, Italy) and the Serodia^®^ HTLV-I particle agglutination assay (Fujirebio, Tokyo, Japan), and confirmed by Western blot by the National Reference Laboratory, Victoria, Melbourne, Australia.

### 2.4. Genomic DNA Isolation

Frozen PBMCs from the HTLV-1c cohort were thawed, and genomic DNA (gDNA) extraction was performed using a GenElute™ Blood Genomic DNA Kit (NA2020, Merck) according to the manufacturer’s instructions. gDNA was eluted in elution buffer, and quantity and quality were measured with UV spectrophotometry (NanoDrop™ 2000, ThermoFisher Scientific, Scoresby, Australia). gDNA was stored at −20 °C until further analysis.

### 2.5. HTLV-1 Proviral Load Measurement

The HTLV-1c proviral load was measured using droplet digital polymerase chain reaction (ddPCR), as previously described [39], with primers (900 µM) and TaqMan probe (FAM, 250 µM) targeting the *tax* gene (Table 2) and 100 ng of patient PBMC gDNA. DdPCR supermix for probes (no dUTP, 1863024, Bio-Rad, Granville, Australia) and the reference gene ribonuclease P/MRP subunit P30 (RPP30, HEX, dHsaCP2500350, Bio-Rad) were used in the assay. Each sample was run in duplicate, and the ddPCR limit of detection for HTLV-1 PVL was determined to be 65 copies per 10^6^ cells.

### 2.6. Plasmids

Plasmids pDR-NL-Δenv,nef-Luc [40], pNBF_gp62cΔTM_codon-opt_S2, pNBF_gp62c_codon-opt (Pr. Paul Young, School of Chemistry and Molecular Biosciences, Queensland, Australia, generated from the Australian subtype C consensus sequence [17]) and pNBF_gp62a_codon-opt possessing ampicillin resistance were transformed into NEB Stable competent E.coli and were grown in Luria-Bertani medium containing 100 µg/mL of ampicillin (Sapphire Scientific, Redfern, Australia).

### 2.7. Expression and Purification of Env Proteins

A construct expressing soluble HTLV-1c Env truncated prior to the membrane-spanning region (pNBF_gp62cΔTM_codon-opt_S2) was transfected into ExpiCHO cells (Gibco, Grand Island, NY, USA) using an ExpiFectamine CHO Transfection Kit (ThermoFisher Scientific) according to the manufacturer’s instructions. The supernatant was harvested after 6 days and filtered using a 0.22 µm filter to remove cellular debris. Strep2-tagged proteins were isolated by StrepTactin XT affinity purification (IBA Life Sciences, Göttingen, Germany) and further purified using size exclusion chromatography with an Äkta Pure 25 L and a Superose 6 prep grade XK 16/70 column (Cytiva, Mount Waverley, Australia).

### 2.8. SDS-PAGE/Western Blot

Samples were separated by SDS-PAGE under denaturing/reducing conditions using 4–12% precast NuPAGE Bis Tris gels using MOPS buffer (Life Technologies, Carlsbad, CA, USA). Proteins were transferred to PVDF membrane (PerkinElmer, Hopkinton, MA, USA) using Tris-Glycine buffer before the membrane was blocked in 5% (*w*/*v*) skim milk powder in PBST. The membranes were probed with primary antibodies or plasma diluted in 2% (*w*/*v*) skim milk powder in PBST (diluent) for 1 h at room temperature. The membranes were then incubated with HRP-conjugated secondary antibody (goat anti-human IgG (Merck) or goat anti-mouse IgG (Invitrogen, Carlsbad, CA, USA)) diluted 1:2000 in diluent for 1 h at room temperature. Bands were visualized using SuperSignal^®^ West Pico Chemiluminescent Substrate (ThermoFisher Scientific).

### 2.9. ELISA

Env-directed antibodies were detected by using purified soluble Env as the coating antigen in an indirect ELISA. Maxisorp 96-well plates were coated with 1 µg/mL of purified Env overnight in coating buffer (20 mM Tris, 100 mM NaCl, pH 8.8) and washed prior to being blocked with 5% skim milk/PBST for 1 h. HTLV-1c+ plasma titrated from a starting dilution factor of 10 was then added and incubated for 2 h. The wells were washed prior to the addition of goat anti-human IgG Fc:HRP at a dilution of 1:5000 and incubated for 1 h; then, a subsequent wash was performed. The reaction was developed by the addition of SureBlue™ TMB Microwell Substrate (SeraCare, Milford, MA, USA) and stopped by the addition of 1 M H_2_SO_4_. Absorbance was measured at 450 nm, and the data were analyzed using a four-parameter logistic regression with the bottom parameter constrained to 0 (GraphPad Prism v 10.4.1). Binding levels are represented as median half-maximal effective dose (EC_50_).

### 2.10. Peptide ELISA

A peptide library was designed to span the entire HTLV-1c Env glycopeptide, with peptides 15 aa in length and overlapping by 11 aa synthesized (Thermo Fisher Scientific) (Table S1). Costar 96-well vinyl assay microplates (Corning, Corning, NY, USA) were coated with 1 μg/mL of peptide (Thermo Fisher Scientific) overnight in coating buffer and washed prior to being blocked with 5% skim milk/PBST for 1 h. HTLV-1c+ patient plasma samples were added and incubated for 2 h. The wells were washed prior to the addition of goat anti-human IgG Fc:HRP at a dilution of 1:2000 and incubated for 1 h; then, a subsequent wash was performed. The reaction was developed by the addition of SureBlue™ TMB Microwell Substrate (SeraCare) and stopped by the addition of 1 M H_2_SO_4_. Absorbance was measured at 450 nm, and the data were analyzed using GraphPad Prism (v 10.4.1).

### 2.11. RsFcγR Dimer-Binding ELISA

A well-established plate-based bioassay for ADCC/ADCP that measures the interaction of HTLV-1c+ patient plasma IgG with four major human FcγRs (the allelic forms of activating type II FcγR, FcγRIIA-Arg131 and FcγRIIA-His131, as well as FcγRIIIA Val158 and Phe158) was investigated by using rsFcγR ectodomains as described [33,41]. Briefly, a 96-well Maxisorp flat-bottom plate was coated with 1 µg/mL of soluble recombinant HTLV-1c Env overnight at 4 °C, then washed prior to being blocked for 1 h at 37 °C with PBS + 1 mM ethylenediaminetetraacetic acid (EDTA) and 1% bovine serum albumin (BSA) (PBSE/BSA). Diluted HTLV-1c+ patient plasma samples were added and incubated for 1 h at 37 °C. The wells were washed prior to the addition of rsFcγRs conjugated to biotin, diluted in PBSE/BSA (0.2 mg/mL for FcγRIIA variants and FcγRIIA, 0.1 mg/mL for FcγRIIIA variants), and incubated for 1 h at 37 °C. The wells were washed prior to the addition of Pierce high-sensitivity streptavidin-HRP (#21130, Thermo Fisher Scientific), then incubated for 1 h at 37 °C. Color development and measurement were performed as mentioned above. Results were analyzed using GraphPad Prism (v 10.4.1).

### 2.12. Pseudovirus (PSV) Neutralization Assay

Pseudotyped HTLV-1 particles were produced by co-transfection of HEK-293T cells with a luciferase-expressing HIV reporter plasmid (pDR-NL-Δenv,nef-Luc) and a vector encoding the full-length HTLV-1 Env retaining an intact transmembrane domain. Transfections of *env* plasmids pNBF_gp62c_codon-opt (the codon optimized consensus sequence of Australian subtype C isolates reported in [17]) or pNBF_gp62a_codon-opt (the codon optimized consensus sequence of Japanese subtype A isolates) were performed at a ratio of 3:1 backbone:env plasmid using Lipofectamine 2000 (Thermo Fisher Scientific) according to the manufacturer’s instructions. After 48 h, culture media were collected and centrifuged for 5 min at 500× *g* before being filtered through a 0.45 µm filter (Sartorius, Göttingen, Germany). Viral particles were then concentrated in a Vivaspin 20 centrifugal concentrator MWCO 100 kDa (Sigma-Aldrich, St. Louis, MO, USA) before being aliquoted and stored at −80 °C.

To test the neutralizing ability of plasma samples, HEK-293T cells were seeded in 96-well flat-bottom plates (Corning). Twenty-four hours later, heat-inactivated plasma samples at a starting dilution of 1:50 were serially diluted in Optimem Reduced Serum Medium (Thermo Fisher Scientific) and incubated with an equal volume of pNL.Luc-gp62co PSV (100,000 RLU), creating a plasma starting dilution of 1:100, for 1 h at 37 °C. The virus/plasma mixture was then added to HEK293T cells and spinoculated for 2 h at 1500× *g* at 20 °C. After 48 h of incubation at 37 °C, Britelite plus Reporter Gene Assay System Reagent (PerkinElmer) was added and incubated for 5 min at room temperature before the luminescence was recorded using the FLUOstar Omega plate reader and Omega version 5.11 software (BMG Labtech, Ortenberg, Germany). Results are represented as residual infectivity relative to a virus-only control and analyzed using a three-parameter logistic regression with the top and bottom parameters constrained to 100 and 0, respectively (GraphPad Prism v 10.4.1). Binding levels are represented as median 50% inhibitory dose (ID_50_), and samples were determined positive for neutralizing activity if they displayed ID_50_ values of >100. Duplicate assays were performed, and if a >2-fold difference was observed in ID_50_ values between experiments, the assay was repeated, and the average of all ID_50_ values was represented.

## 3. Results

### 3.1. Individuals Confirmed with HTLV-1c Infection Commonly Develop Env-Directed Ab Responses

**Figure 1:**
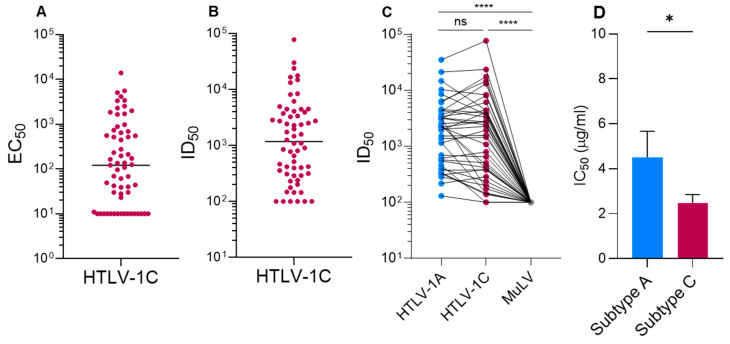
Binding and neutralizing activity of diagnostically confirmed HTLV-1c+ plasma IgG. (**A**) Binding of plasma IgG from a First Nations cohort from Central Australia to recombinant Env in an indirect ELISA. Limit of detection = 10; results are represented by EC_50_ values. (**B**) Neutralization of NL.luc-gp62c PSV by plasma IgG. Limit of detection = 100; results are represented by ID_50_ values (**C**) Neutralizing activity of a subset (n = 40) of plasma samples comparing neutralizing titers against NL.luc-gp62a, NL.luc-gp62c and NL.luc-MuLV. (**D**) Neutralizing titers (µg/mL) of the rat nAb LAT-27 against NL.luc-gp62a and NL.luc-gp62c. Samples were tested in duplicate in at least 2 independent experiments with internal positive and negative control samples. Statistical analysis performed using the Kruskal–Wallis test; **** = *p*-value < 0.0001, * = *p*-value < 0.05, and ns = not significant. Values represent the mean of a minimum of two independent experiments, and error bars represent SEM.

The prevalence of anti-Env responses in individuals from an HTLV-1c+ cohort (n = 62) based at the Alice Springs Hospital (ASH), Central Australia, was assessed using paired plasma and PBMC samples. Plasma was tested for binding to soluble HTLV-1c Env protein by indirect ELISA (Figure 1A). Of the 62 diagnosed HTLV-1-positive plasma samples tested, 76% (47/62) displayed binding to soluble Env (median half-maximal effective dose (EC_50_) = 115, 95% CI: 40–232), indicative of seroconversion in the majority of infected individuals.

### 3.2. Anti-Env Responses Exhibit Neutralizing Activity and Breadth in HTLV-1c+ Individuals

Antibodies directed against Env are well documented in their ability to block viral entry by binding and occluding epitopes that are essential for host cell receptor docking and, consequently, viral entry. The ability of plasma IgG to prevent viral entry was assessed using a PSV neutralization assay utilizing pseudotyped particles expressing a luciferase reporter and full-length HTLV-1c Env (NL.luc-gp62c). Of the 62 samples tested, 90% showed varying levels of neutralization (median 50% inhibitory dose (ID_50_) = 1173, 95% CI: 463–2499) (Figure 1B). To examine neutralization breadth, a subset of these samples (n = 40) was examined for neutralizing activity against PSV expressing subtype-A Env (NL.luc-gp62a) (Figure 1C). The mean neutralization titer was 5436 and 4058 against NL.luc-gp62a and NL.luc-gp62c, respectively, with no statistically significant difference between the groups, indicating a cross-neutralizing response. Interestingly, some samples demonstrated greater neutralizing titers against NL.luc-gp62a compared with NL.luc-gp62c. LAT-27 showed neutralizing activity against both PSVs, with an IC_50_ of 4.5 µg/mL against subtype A, consistent with what has been reported (Figure 1D) [42]. Unexpectedly, LAT-27 showed greater potency against subtype C, with an IC_50_ of 2.5 µg/mL (*p*-value < 0.0385, 95% CI: 0.118–3.936). As expected, all samples tested failed to neutralize the unrelated Murine Leukemia virus pseudotyped particles (NL.luc-MuLV), demonstrating the specificity of the assay for HTLV-1.

### 3.3. Mapping of Ab Binding Epitopes from HTLV-1c+ Individuals

**Figure 2:**
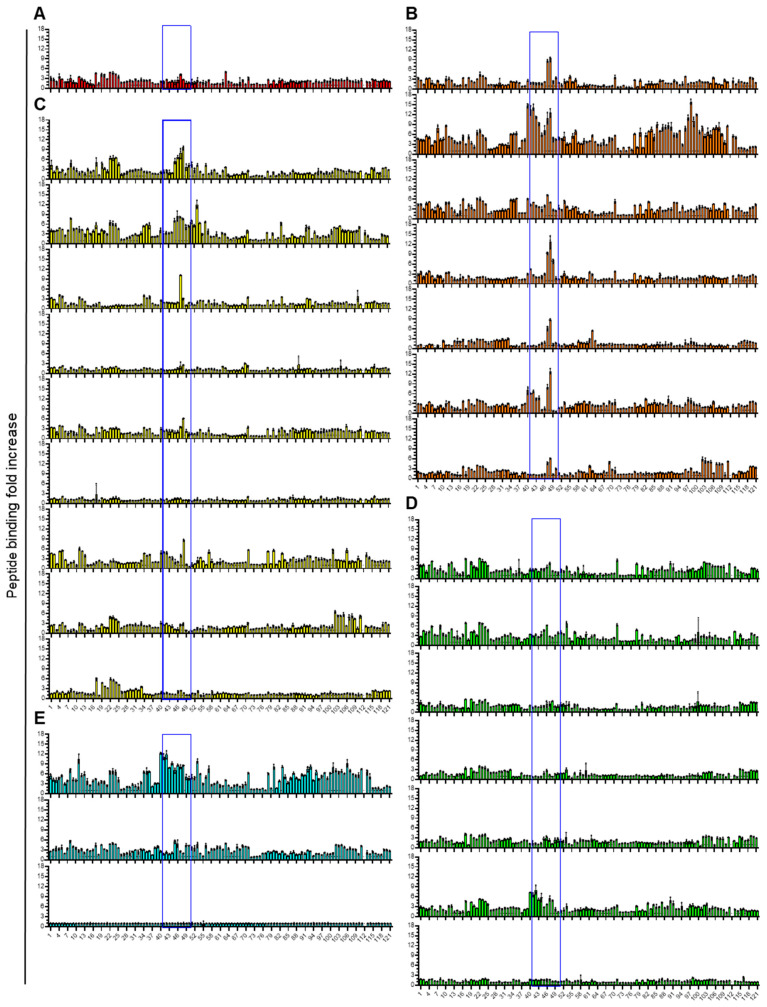
Mapping of anti-HTLV-1c+ Env IgG binding epitopes. An Env-spanning peptide library was used to identify common binding epitopes in an indirect ELISA. Coating peptide IDs are listed on the x-axis; donor plasma samples were diluted 1:100. Fold change was calculated by dividing the O.D. of HTLV-1c+ samples by the O.D. of the HTLV-1c- sample control. Graphs color-coded according to (**A**) very high—red, (**B**) high—orange, (**C**) intermediate—yellow, (**D**) low—green and (**E**) negative—blue neutralizers. Values represent the mean of triplicate experiments; error bars = standard deviation. Blue box: proline-rich region (PRR).

**Figure 3:**
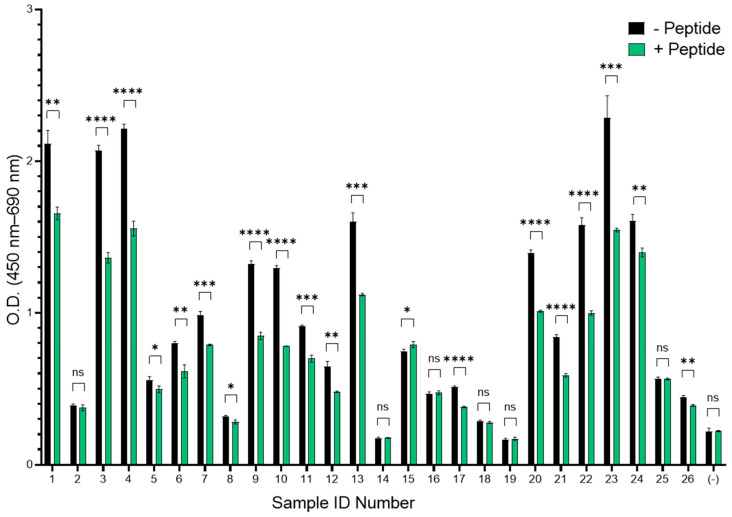
Competitive ELISA with HTLV-1c synthetic Env peptides reduces binding of HTLV-1c+ patient plasma IgG to soluble HTLV-1c Env protein. Donor plasma samples (n = 26) were diluted 1:100 and preincubated with synthetic peptides 47 and 48 at a concentration of 10 µg/mL. Black: without peptides; grey: with peptides. Error bars represent standard deviation; **** = *p*-value < 0.0001, *** = *p*-value < 0.001, and ** = *p*-value < 0.01. * = *p*-value < 0.05. ns = not significant. (-): uninfected plasma.

The Env binding epitopes of a subset of patient plasma samples (n = 26) were mapped using a library of synthetic peptides designed to span the full Env glycoprotein sequence of HTLV-1c. Peptides 15 aa in length and overlapping by 11 aa were synthesized and used as the binding antigen in an indirect peptide ELISA. Overall, binding of plasma antibodies to the peptide library varied between HTLV-1c+ individuals (Figure 2). However, a peak in binding was observed across peptides 47–49, corresponding to the PRR, a well-documented binding epitope for neutralizing antibodies [29,30] in the majority of samples that were categorized as high and intermediate neutralizers (Figure 2A,B). Importantly, when these plasma samples were preincubated with peptides 47 (PTAPPLLPHSNLDHI) and 48 (PLLPHSNLDHILEPS), which span the LAT-27 binding epitope located in the PRR, binding to soluble Env was significantly reduced in 77% of the samples (Figure 3). This suggests that PRR-directed Ab responses developed in most donors tested.

### 3.4. Correlation Between Anti-Env Responses and PVL

**Figure 4:**
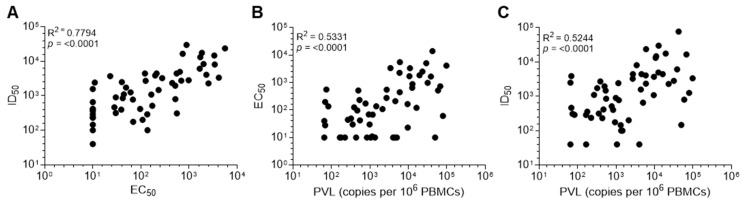
Correlation between PVL and Env-directed Ab levels. Correlation between (**A**) HTLV-1c+ donor plasma IgG neutralizing (ID_50_) and binding (EC_50_) activity, (**B**) HTLV-1c+ donor plasma IgG EC_50_ and PVL and (**C**) HTLV-1c+ donor plasma IgG ID_50_ and PVL. Statistical analysis performed using Spearman’s correlation test.

The correlation between the Env-specific (EC_50_) and neutralizing (ID_50_) Ab responses in HTLV-1c+ plasma was assessed. Indeed, a high positive correlation (R_2_ = 0.7794, *p*-value < 0.0001, 95% CI: 0.6534–0.8634) was observed (Figure 4A and Figure S1), showing the high frequency of nAb development during natural infection. Previous studies have reported that PVL strongly correlates with seropositivity against pooled viral structure antigens in subtype-A infection, but there have been conflicting reports about correlations with Env-specific Ab levels [35,38,43,44]. The PVL in PBMCs from these donors was measured using droplet digital PCR (ddPCR) with primer/probes targeting the *tax* gene of the integrated provirus [39]. A strong positive correlation between PVL and both EC_50_ (*p*-value < 0.0001, 95% CI: 0.3116–0.6996) and ID_50_ (*p*-value < 0.0001, 95% CI: 0.2984–0.6947) was observed (Figure 4B,C).

### 3.5. Correlation Between Anti-Env Responses and Clinical Disease

**Figure 5:**
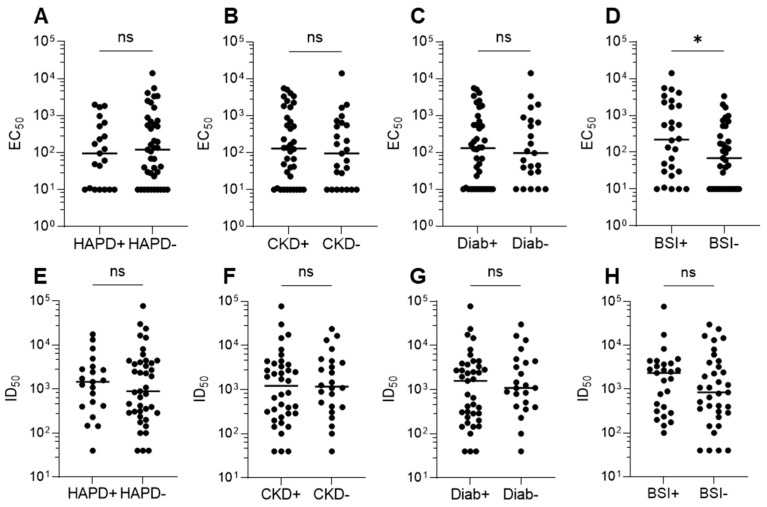
Examination of the relationship between disease association and Env-specific Ab responses. (**A**–**D**) Env-targeting Ab titers were compared in individuals with and without recorded HTLV-1-associated pulmonary disease (HAPD), chronic kidney disease (CKD), diabetes (Diab) or a history of blood stream infection (BSI). (**E**–**H**) Neutralizing Ab titers were also compared in these groups. * = *p*-value < 0.05. ns = not significant.

Any association of anti-Env IgG titers with clinical disease was examined by comparison of EC_50_ and ID_50_ between HTLV-1c+ individuals diagnosed with HTLV-1-associated pulmonary disease (HAPD), chronic kidney disease (CKD), diabetes (Diab) or recorded blood stream infections (BSI) and those who were not. No significant association was observed between Env-specific binding titers and HAPD, CKD and diabetes; however, EC_50_ was significantly higher in the BSI+ group compared to the BSI- group (*p*-value < 0.0235, 95% CI: 166–2213) (Figure 5A–D). A positive trend with comorbidity and ID_50_ was observed in all groups, albeit not statistically significant (Figure 5E–H). No correlation of EC_50_ or ID_50_ was observed with age, an approximate measure of infection duration (Figure S2).

### 3.6. Anti-Env IgG from HTLV-1c+ Individuals Bind FcγRs

**Figure 6:**
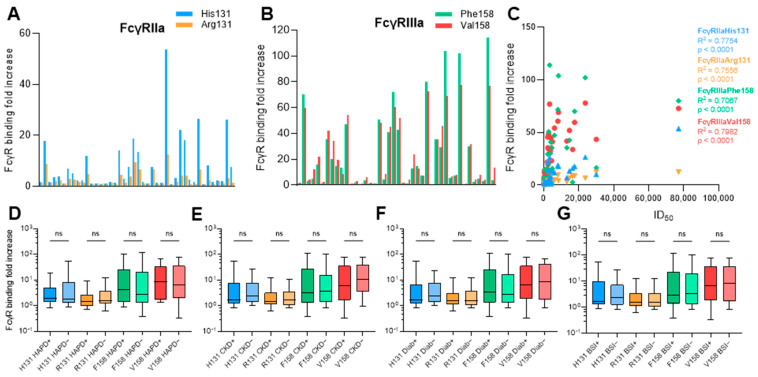
Comparison of FcγR engagement by HTLV-1c+ plasma IgG. Engagement of HTLV-1c+ plasma IgG (diluted 1:100) with (**A**) FcγRIIa and (**B**) FcγRIIIa allotypes. (**C**) Correlation between FcγR binding and neutralizing activity (ID_50_). Samples tested in duplicate in at least 2 independent experiments. Associations of FcγR binding with (**D**) HAPD, (**E**) CKD, (**F**) diabetes and (**G**) BSI were also assessed. ns = not significant.

The interaction between IgGs and FcγRs expressed on human immune cells is an important determinant of the ability to mediate Ab effector functions such as ADCC and ADCP. The binding of plasma IgG to two allotypes of FcγRIIa (H131 and R131) and FcγRIIIa (F158 and V158) soluble dimeric human FcγRs (sFcγRs) using indirect ELISA [33] was assessed. HTLV-1c+ plasma IgG bound both FcγRIIa allotypes tested to similar extents (33% and 35% of samples bound the H131 and R131 allotypes, respectively) (Figure 6A). Plasma IgG strongly engaged sFcγRIIIa V158, with 61% of clinical samples positive for binding compared with the F158 allotype (35%) (Figure 6B). Unexpectedly, engagement of all FcγRs positively correlated with neutralizing activity (*p*-values < 0.0001, 95% CI H131: 0.6475–0.8608, R131: 0.6188–0.8479, F158: 0.5496–0.8155, V158: 0.6809–0.8755) (Figure 6C). This suggests that anti-Env responses arising during natural infection can activate immune effector functions, particularly ADCC. There was no significant association with disease (HAPD, CKD, diabetes or BSI) for either of the FcγRIIa or FcγRIIIa allotypes (Figure 6D–G).

## 4. Discussion

This study assessed the potency, specificity and breadth of Env-directed adaptive immune responses that arise during natural HTLV-1c infection in an Australian First Nations cohort with the aim of informing future Env-based vaccine design efforts. The rate of Env-directed nAb induction was high, with the majority of HTLV-1c+ individuals tested demonstrating the presence of neutralizing activity against both subtype-A and subtype-C, a clear indication of breadth. All samples tested failed to neutralize the unrelated MuLV PSV, demonstrating the specificity of testing for HTLV-1 Env. Env-binding responses were observed in 75% of samples, consistent with previous reports of subtype-A infection [45]. This substantiates the premise that Env presented on infected cells and virions as an antigen can induce robust protective immunity against HTLV-1 and suggests that the recombinant soluble Env protein used in our ELISA assays does not display all immunodominant neutralizing epitopes. This supports the focus on native-structured Env trimers as vaccine targets for antibodies. The ability of plasma from HTLV-1c+ donors to neutralize both subtype-A and subtype-C PSV also highlights the possibility that developing a universal vaccine is achievable. The observed higher neutralization titers against subtype-A PSV in some donors infected with subtype-C were unexpected, as sequencing analysis has shown that the predominant circulating subtype in Central Australia is almost exclusively subtype C [17]. The ddPCR assay used in this study can discriminate between subtypes, and the cohort showed no evident subtype-A infections. Although co-infection with both subtypes cannot be ruled out, the characteristics of uniform, broad and potent neutralizing antibodies may relate to high genetic similarities in these closed community groups.

The discrepancy between some samples in ELISA and PSV binding activity may be explained by a number of reasons: the major Env-specific neutralizing epitopes for these donors are conformationally sensitive and are not presented by wild-type soluble recombinant Env, the sensitivity of the ELISA is unable to measure the low-affinity binding of some samples, or potential non-specific neutralizing activity. The failure of any samples to neutralize MuLV PSV suggests the latter is not the case, and further investigation with improved prefusion-stabilized Env antigens will elucidate this point. The PRR of gp46 is a major neutralizing domain of the Env glycoprotein [46] and LAT-27, one of the few well-characterized HTLV-1 nAbs, targets the residues Leu-Pro-His-Ser-Asn-Leu at aa 191–196 located within the PRR [29,30]. We showed that this epitope is also commonly targeted by plasma IgG of HTLV-1c+ individuals, particularly in high neutralizers, suggesting that this is also an important immunogenic epitope during subtype-C infection. Passive immunization of LAT-27 in a humanized mouse model at 10 mg/kg prevented viral replication and disease, making it a clear correlate of protection in vivo [31]. Authentic presentation of this epitope will therefore be a vital consideration for future vaccine design efforts, as well as therapeutic mAb treatment development [47].

During natural HTLV-1 infection, the detection of viral antigen-expressing cells in peripheral blood is low, and the role of nAb in controlling infection progression is poorly understood. Any association between PVL and nAb levels is unclear, as a previous report observed a positive correlation between nAb and PVL in HTLV-1a-infected ACs [44]; however, another report observed no correlation in ACs or ATL patients [35]. Here, we have demonstrated a positive correlation between PVL and anti-Env seropositivity [48], indicating that anti-Env Ab responses do not function to control infection progression. However, some donors showed high PVL but low binding and/or neutralizing titers, and vice versa, highlighting the complex interplay between virus and host during prolonged infection and antigen exposure. Previous studies have identified defective proviruses where the 5′ LTR is commonly deleted, with the 3′ LTR and antisense *hbz* remaining largely intact [49,50]. Large internal deletions encoding viral structural genes such as Env have been identified in HTLV-1c+ individuals in Australia, yet it is unknown how this affects the development of Abs targeting these proteins [17]. The current study suggests that an HTLV-1 reservoir expressing intact Env is maintained during infection, which allows for long-term exposure to Env antigen and is responsible for maintaining the potent nAb responses observed in this study. Recapitulating this antigen exposure in a vaccination regimen may prove challenging and will likely require multiple boosts to generate similar levels of nAbs.

The findings from this study suggest that testing serology titers in Central Australia could provide a correlate for the prediction of PVL in HTLV-1c+ individuals, which is a demonstrated predictor of disease severity [5,36,37]. This would support care of at-risk communities in the remote settings of Central Australia, where all confirmatory PVL testing is performed interstate and delayed results can affect patient follow-up care and support. The correlation between HTLV-antibody titer and PVL also suggests that Env-targeting antibodies do not control infection once it is established. No association of Env-directed Abs with HAPD, diabetes or CKD was observed, suggesting Ab levels will not be useful as a biomarker or therapeutic for disease treatment in general. This was also the case in subtype-A infection, where Env-specific Ab levels did not correlate with PVL in ATL and HAM patients [19,20]. In the current study, we observed a positive association of EC_50_ with a history of BSIs. First Nations communities of Central Australia experience some of the highest rates of BSIs globally, which are associated with HTLV-1c PVL, serological diagnosis and infection with the human parasite *Strongyloides stercoralis* [51,52,53]. These confounding factors present challenges for the analysis of a relationship between clinical BSIs and Env-directed Ab responses in this cohort; therefore, further investigation will be required.

ADCP and ADCC are important defense mechanisms against viral infection, and high ADCC-inducing Abs have previously been identified in individuals with HTLV-1a infection [54,55,56]. Here, we used a robust and high-throughput dimeric FcγR ELISA-based assay to detect HTLV-1-specific Abs with ADCP and ADCC potential [33,57]. We detected both FcγRIIa and FcγRIIIa dimer engagement by HTLV-1c+ plasma IgG, indicative of the potential for both ADCP and ADCC in this cohort. A greater proportion of samples (61%) were positive for FcγRIIIa dimer engagement, suggesting an increased propensity for ADCC activity in vivo. Future studies with cell-based assays will be used to confirm functional ADCP and ADCC activity in HTLV-1c-infected plasma samples [56]. A study by Tanaka et al. showed no association between ADCC activity and disease in patients with acute ATL and asymptomatic carriers, and, similarly, we observed no association of FcγR binding with HAPD, diabetes, CKD or BSI, which have been identified as correlates of HTLV-1c infection in Australia [35]. This suggests that although infected individuals can generate humoral responses after infection, they are not sufficient to control disease progression. Concurrently, the virus employs a suite of immune evasion strategies, including cell-to-cell infection, downregulation of productive infection and T cell clonal expansion driven by antisense *hbz* RNA and poorly immunogenic HBZ protein [58].

Due to the remote nature of sampling from Central Australia and the difficulties encountered in processing clinical samples in this setting, acquiring large numbers of viable matched PBMCs and plasma is challenging. However, even with these limitations, we achieved an in-depth assessment of the humoral responses to HTLV-1c infection, demonstrating that infected individuals in these closed communities regularly produce robust and neutralizing immunity to HTLV-1 during infection. This suggests that a recombinant Env-based vaccine could protect First Nation Australians from HTLV-1c infection, reducing the extremely high infection rates recorded in Central Australia and the HTLV-1c-associated disease burden.

## Figures and Tables

**Table 1:** Demographic data for 62 HTLV-1c+ Australian First Nations participants.

Characteristic	Cases (*n* = 62)
Age, years (mean ± SD)	54.4 ± 12.7
Sex (n, %)	Men = 22 (34.9) Women = 40 (64.5)
Comorbidities (n, %)	
Diabetes	39/62 (62.9)
Asthma	5/62 (8.1)
IHD	14/62 (22.6)
CLD	10/62 (16.1)
CKD	38/62 (61.3)
Possible HTLV-1-associated conditions (n, %)	
Bronchiectasis	20/62 (32.3)
COPD	5/62 (8.1)
BSI *	28/62 (45.2)
Strongyloidiasis seropositive	11/61 (18.0) ^Ø^
Lifestyle (n, %)	
Smoking history	21/55 (38.2) ^Ø^
Alcohol history	28/58 (48.3) ^Ø^

Abbreviations: BSI—blood stream infection; CLD—chronic liver disease; CKD—chronic kidney disease; COPD—chronic obstructive pulmonary disease; IHD—ischemic heart disease. Missing data are indicated with ^Ø^. * current or previous BSI identified from microbiological records. Bex—bronchiectasis, HAPD—HTLV-1- associated pulmonary disease, defined as participants diagnosed with Bex or COAD/COPD (chronic obstructive airways disease/chronic obstructive pulmonary disease), and BSI—blood stream infection.

**Table 2:** Primers and probes used for quantification of HTLV-1c and T cells by ddPCR.

Name	Target	Sequence (5′-3′)
ODP 3085 (forward)	HTLV-1c *tax* region	TCCAGGCCTTATTTGGACAT
ODP 3086 (reverse)	HTLV-1c *tax* region	CGTGTGAGAGTAGGACTGAG
ODP 3318 *tax* probe	HTLV-1c *tax* region	6FAM-CATGATTTCCGGGCCTTGC-MGBNFQ *

* 6FAM—5′ fluorescent dye; MGBNFQ—3′ minor groove binding non-fluorescent quencher.

## Data Availability

The original contributions presented in this study are included in the article. Further inquiries can be directed to the corresponding author.

## Supplementary Materials

The following supporting information can be downloaded at https://www.mdpi.com/article/10.3390/v18040402/s1: Figure S1: Heatmap of participant EC_50_ and ID_50_ values; Figure S2: Correlation of study participant age at recruitment with EC_50_ and ID_50_; Table S1: HTLV-1c peptide library sequences.

## Abbreviations

The following abbreviations are used in this manuscript:

ADCC	Antibody-dependent cellular cytotoxicity
ADCP	Antibody-dependent cellular phagocytosis
ATL	Adult T-cell leukemia
CKD	Chronic kidney disease
ddPCR	Droplet digital PCR
GLUT1	Glucose transporter type 1
HAM	HTLV-1-associated myelopathy
HAPD	HTLV-1-associated pulmonary disease
HTLV-1	Human T-cell leukemia virus type-1
HSPG	Heparin sulphate proteoglycans
MTCT	Mother-to-child-transmission
NRP1	Neuropilin 1
PRR	Proline rich region
PVL	Proviral load
