# Supplementary material for: Common Acquisition of Broadly Neutralizing Antibodies in an HTLV-1c+ First Nations Cohort from Central Australia
Source: Viruses. 2026 Mar 24;18(4):402. doi: 10.3390/v18040402 (PMC13119588; doi:10.3390/v18040402)

**Table S1:** HTLV-1c peptide library sequences.

| Peptide No. | Sequence | Env Subunit | Features |
|---|---|---|---|
| 1[2] | MGKFLTTLILFLQFC | gp46 | |
| 2[2] | LTTLILFLQFCPPIL | gp46 | |
| 3 | ILFLQFCPPILCYYS | gp46 | |
| 4[2] | QFCPPILCYYSPSCC | gp46 | |
| 5[2] | PILCYYSPSCCTLTI | gp46 | |
| 6[2] | YYSPSCCTLTIGVSS | gp46 | |
| 7 | SCCTLTIGVSSYHSK | gp46 | |
| 8 | LTIGVSSYHSKPCNP | gp46 | |
| 9 | VSSYHSKPCNPAQPV | gp46 | |
| 10 | HSKPCNPAQPVCSWT | gp46 | |
| 11[2] | CNPAQPVCSWTLDLL | gp46 | |
| 12[2] | QPVCSWTLDLLALSA | gp46 | |
| 13[2] | SWTLDLLALSADQAL | gp46 | |
| 14 | DLLALSADQALQPPC | gp46 | |
| 15 | LSADQALQPPCPNLV | gp46 | |
| 16 | QALQPPCPNLVSYSN | gp46 | |
| 17 | PPCPNLVSYSNYHAT | gp46 | |
| 18[2] | NLVSYSNYHATYSLY | gp46 | |
| 19 | YSNYHATYSLYLFPH | gp46 | |
| 20 | HATYSLYLFPHWIKK | gp46 | |
| 21 | SLYLFPHWIKKPNRN | gp46 | NRP-1 binding site |
| 22 | FPHWIKKPNRNGGGY | gp46 | NRP-1 binding site |
| 23 | IKKPNRNGGGYYSAS | gp46 | NRP-1 binding site |
| 24 | NRNGGGYYSASYSDP | gp46 | |
| 25 | GGYYSASYSDPCSLK | gp46 | |
| 26 | SASYSDPCSLKCPYL | gp46 | |
| 27 | SDPCSLKCPYLGCQS | gp46 | |
| 28 | SLKCPYLGCQSWTCP | gp46 | |
| 29 | PYLGCQSWTCPYTGA | gp46 | |
| 30 | CQSWTCPYTGAVSSP | gp46 | |

| | | | |
|---|---|---|---|
| 31 | TCPYTGAVSSPYWKF | gp46 | |
| 32 | TGAVSSPYWKFQQDV | gp46 | |
| 33 | SSPYWKFQQDVNFTQ | gp46 | |
| 34[2] | WKFQQDVNFTQEVSR | gp46 | |
| 35[2] | QDVNFTQEVSRLNIN | gp46 | |
| 36[2] | FTQEVSRLNINLHFS | gp46 | |
| 37[2] | VSRLNINLHFSKCGF | gp46 | |
| 38 | NINLHFSKCGFPFSL | gp46 | |
| 39 | HFSKCGFPFSLLVDA | gp46 | |
| 40 | CGFPFSLLVDAPGYD | gp46 | Proline Rich Region |
| 41 | FSLLVDAPGYDPIWL | gp46 | Proline Rich Region |
| 42 | VDAPGYDPIWLLNTE | gp46 | Proline Rich Region |
| 43 | GYDPIWLLNTEPSQL | gp46 | Proline Rich Region |
| 44 | IWLLNTEPSQLPPTA | gp46 | Proline Rich Region |
| 45 | NTEPSQLPPTAPPLL | gp46 | Proline Rich Region |
| 46 | SQLPPTAPPLLPHSN | gp46 | Proline Rich Region |
| 47 | PTAPPLLPHSNLDHI | gp46 | Proline Rich Region |
| 48 | PLLPHSNLDHILEPS | gp46 | Proline Rich Region |
| 49 | HSNLDHILEPSIPWK | gp46 | Proline Rich Region |
| 50 | DHILEPSIPWKSKLL | gp46 | Proline Rich Region |
| 51 | EPSIPWKSKLLTLVQ | gp46 | Proline Rich Region |
| 52 | PWKSKLLTLVQLTLQ | gp46 | Proline Rich Region |
| 53 | KLLTLVQLTLQSTNY | gp46 | Proline Rich Region |
| 54[2] | LVQLTLQSTNYTCIV | gp46 | Proline Rich Region |
| 55[2] | TLQSTNYTCIVCIDR | gp46 | |
| 56 | TNYTCIVCIDRASLS | gp46 | |
| 57[2] | CIVCIDRASLSTWHV | gp46 | |
| 58 | IDRASLSTWHVLYSP | gp46 | |
| 59 | SLSTWHVLYSPNISI | gp46 | |
| 60 | WHVLYSPNISIPSSS | gp46 | |
| 61 | YSPNISIPSSSSTPL | gp46 | |
| 62 | ISIPSSSSTPLLYPS | gp46 | |

| | | | |
|---|---|---|---|
| 63 | SSSSTPLLYPSLALP | gp46 | |
| 64 | TPLLYPSLALPAPHL | gp46 | |
| 65 | YPSLALPAPHLTLPF | gp46 | |
| 66 | ALPAPHLTLPFNWTH | gp46 | |
| 67 | PHLTLPFNWTHCFDP | gp46 | |
| 68 | LPFNWTHCFDPQIQA | gp46 | |
| 69 | WTHCFDPQIQAIVSS | gp46 | |
| 70 | FDPQIQAIVSSPCHN | gp46 | |
| 71[2] | IQAIVSSPCHNSLIL | gp46 | |
| 72 | VSSPCHNSLILPPFS | gp46 | |
| 73 | CHNSLILPPFSLSPV | gp46 | |
| 74 | LILPPFSLSPVPTLR | gp46 | |
| 75 | PFSLSPVPTLRSRSR | gp46 | |
| 76 | SPVPTLRSRSRRAVP | gp46/gp21 | Fusion Peptide |
| 77 | TLRSRSRRAVPVAVW | gp46/gp21 | Fusion Peptide |
| 78[2] | RSRRAVPVAVWLVSA | gp46/gp21 | Fusion Peptide |
| 79 | AVPVAVWLVSALAMG | gp46/gp21 | Fusion Peptide |
| 80[2] | AVWLVSALAMGTGIA | gp21 | Fusion Peptide |
| 81 | VSALAMGTGIAGGIT | gp21 | Fusion Peptide |
| 82 | AMGTGIAGGITGSMS | gp21 | Fusion Peptide |
| 83[2] | GIAGGITGSMSLASG | gp21 | Fusion Peptide |
| 84 | GITGSMSLASGKNLL | gp21 | Fusion Peptide |
| 85 | SMSLASGKNLLHEVD | gp21 | |
| 86 | ASGKNLLHEVDKDIS | gp21 | |
| 87 | NLLHEVDKDISQLTQ | gp21 | |
| 88 | EVDKDISQLTQAIVK | gp21 | |
| 89 | DISQLTQAIVKNHKN | gp21 | |
| 90 | LTQAIVKNHKNLLKI | gp21 | |
| 91 | IVKNHKNLLKIAQYA | gp21 | |
| 92[2] | HKNLLKIAQYAAQNR | gp21 | |
| 93[2] | LKIAQYAAQNRRGLD | gp21 | |
| 94 | QYAAQNRRGLDLLFW | gp21 | |

| | | | |
|---|---|---|---|
| 95[2] | QNRRGLDLLFWEQGG | gp21 | |
| 96 | GLDLLFWEQGGLCKA | gp21 | |
| 97 | LFWEQGGLCKALQEQ | gp21 | |
| 98 | QGGLCKALQEQCCFL | gp21 | |
| 99 | CKALQEQCCFLNITN | gp21 | |
| 100 | QEQCCFLNITNSHVS | gp21 | |
| 101[2] | CFLNITNSHVSILQE | gp21 | |
| 102 | ITNSHVSILQERPPL | gp21 | |
| 103 | HVSILQERPPLENRV | gp21 | |
| 104 | LQERPPLENRVLTGW | gp21 | |
| 105 | PPLENRVLTGWGLNW | gp21 | |
| 106[2] | NRVLTGWGLNWDLGL | gp21 | |
| 107 | TGWGLNWDLGLSQWA | gp21 | |
| 108 | LNWDLGLSQWAREAL | gp21 | |
| 109 | LGLSQWAREALQTGI | gp21 | Transmembrane Region |
| 110[2] | QWAREALQTGITLVA | gp21 | Transmembrane Region |
| 111 | EALQTGITLVALLLL | gp21 | Transmembrane Region |
| 112[1] | TGITLVALLLLVILA | gp21 | Transmembrane Region |
| 113[2] | LVALLLLVILAGPCI | gp21 | Transmembrane Region |
| 114[2] | LLLVILAGPCILRQL | gp21 | Transmembrane Region |
| 115 | ILAGPCILRQLRQLP | gp21 | |
| 116 | PCILRQLRQLPSRTR | gp21 | |
| 117 | RQLRQLPSRTRYPHY | gp21 | |
| 118 | QLPSRTRYPHYSLIN | gp21 | |
| 119 | RTRYPHYSLINPESS | gp21 | |
| 120 | TRYPHYSLINPESSL | gp21 | |

[1]Unable to be synthesised

[2]Lysophilized and resuspended in DMSO

**Figure S1:** Heatmap of participant $EC_{50}$ and $ID_{50}$ values.

| Donor Sample | EC50 | ID50 |
|---|---|---|
| DY9 | 884 | 30045 |
| DY10 | 68 | 774 |
| DY11 | 44 | 1096 |
| DY12 | <10 | 288 |
| DY13 | 42 | 848 |
| DY14 | <10 | <100 |
| DY15 | 644 | 4929 |
| DY16 | 121 | 4451 |
| DY17 | 30 | 898 |
| DY18 | 49 | 1726 |
| DY19 | 40 | 2499 |
| DY20 | <10 | 356 |
| DY21 | 61 | 1250 |
| DY22 | 446 | 2347 |
| DY23 | <10 | <100 |
| DY24 | <10 | <100 |
| DY26 | 275 | 3238 |
| DY27 | <10 | 230 |
| DY29 | 734 | 16514 |
| DY30 | 30 | 315 |
| DY31 | <10 | <100 |
| P102 | 4133 | 3344 |
| P034 | 137 | <100 |
| P029 | 1843 | 17618 |
| P008 | <10 | 144 |
| P032 | 556 | 309 |
| P125 | 515 | 1963 |
| P143 | 28 | 463 |
| P054 | 1704 | 13232 |
| P072 | 688 | 2726 |
| P078 | 11 | 2419 |
| P141 | 164 | 1084 |
| P070 | 1655 | 6127 |
| P002 | <10 | <100 |
| P094 | 211 | 4437 |
| P067 | 39 | 395 |
| P098 | 135 | 289 |
| P077 | 125 | 2724 |
| P091 | 96 | 415 |
| P127 | 975 | 2830 |
| P136 | 517 | 799 |
| P086 | 2276 | 4061 |
| P082 | 3394 | 8094 |
| P021 | 3332 | 14744 |
| P030 | 232 | 1460 |
| P140 | 172 | 511 |
| P033 | <10 | 400 |
| P103 | 108 | 201 |
| P085 | 13997 | 77060 |
| P138 | 69 | 176 |
| P025 | 549 | 4419 |
| P066 | 2523 | 2284 |
| P158 | 1995 | 8356 |
| P045 | <10 | 652 |
| P143 | 197 | 3892 |
| P046 | <10 | 1578 |
| P146 | 5555 | 23724 |
| P039 | <10 | 144 |
| P095 | 23 | 3708 |
| P040 | <10 | 237 |
| P058 | <10 | 418 |
| P015 | <10 | 147 |

| | | | | | |
|---|---|---|---|---|---|
| **ID50** | ≤100 | 101 - 500 | 501 - 1500 | 1501 - 3000 | >3001 |
| **EC50** | ≤10 | 11 - 250 | 251 - 500 | 501 - 1000 | >1000 |

**Figure S2:** Correlation of study participant age at recruitment with (**a**) $EC_{50}$ and (**b**) $ID_{50}$.

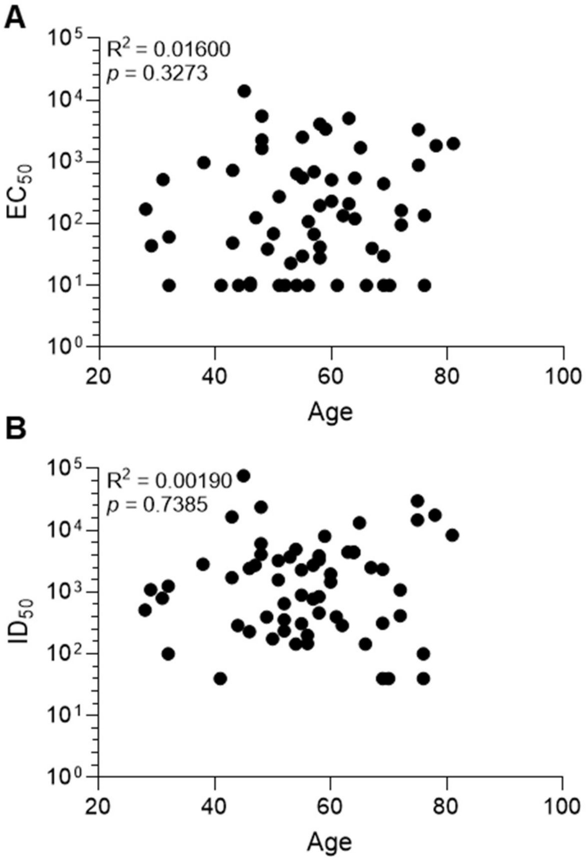

Supplement: Supplementary file 1 [file viruses-18-00402-s001.zip › viruses-4196618-supplementary.pdf]
